# Vectorial transport of the arginine derivatives asymmetric dimethylarginine (ADMA) and l-homoarginine by OATP4C1 and P-glycoprotein studied in double-transfected MDCK cells

**DOI:** 10.1007/s00726-020-02867-8

**Published:** 2020-07-08

**Authors:** Emir Taghikhani, Renke Maas, R. Verena Taudte, Arne Gessner, Martin F. Fromm, Jörg König

**Affiliations:** Institute of Experimental and Clinical Pharmacology and Toxicology, Friedrich-Alexander-Universität Erlangen-Nürnberg, Fahrstrasse 17, 91054 Erlangen, Germany

**Keywords:** Proximal tubule cells, Uremic toxin, ADMA, l-Homoarginine, SLC transporter, OATP4C1, *SLCO4C1*

## Abstract

Elevated plasma concentrations of the uremic toxin asymmetric dimethylarginine (ADMA) and low plasma concentrations of l-homoarginine are independently associated with cardiovascular events and mortality. Key enzymes involved in the homeostasis of both arginine derivatives are expressed in proximal tubule cells of the kidney. To get access to these enzymes, transport proteins are important. One of the transporters mediating the transport of ADMA and l-homoarginine is the solute carrier superfamily (SLC) member OATP4C1, located in the basolateral membrane of proximal tubule cells. To gain insights into the role of export pumps in the transport of both substances, we established a double-transfected MDCK cell line expressing OATP4C1 and the export pump P-glycoprotein (P-gp). Using MDCK cell monolayers, we demonstrated in time-dependent and concentration-dependent vectorial transport experiments that ADMA and l-homoarginine are transported from the basolateral to the apical compartment of MDCK-OATP4C1-P-gp cells with significantly higher transport rates compared to single-transfected MDCK-OATP4C1, MDCK-P-gp and MDCK-VC (control) cells (e.g. transport ratio MDCK-OATP4C1-P-gp/MDCK-VC: for 50 µM ADMA = 2.0-fold, for 50 µM l-homoarginine  = 3.4-fold). These results indicate that both OATP4C1 and P-gp transport the arginine derivatives ADMA and l-homoarginine and are, therefore, important for the homoeostasis of both substances.

## Introduction

The arginine derivative asymmetric dimethylarginine (ADMA) is an endogenously formed byproduct of protein catabolism (Tang 2000) and is possibly involved in the regulation of endothelial function (Vallance et al. 1992b). Various studies reported an association between elevated plasma concentrations of ADMA and an increase of cardiovascular and all-cause mortality (Schlesinger et al. 2016). The elimination of ADMA from the body is achieved via enzymatic degradation by the two enzymes dimethylaminohydrolase 1 (DDAH1) and alanine:glyoxylate aminotransferase 2 (AGXT2), both expressed in renal proximal tubule cells. Accordingly, plasma concentrations of ADMA rise up to threefold in chronic kidney disease (CKD) (Jacobi and Tsao 2008; Vallance et al. 1992a). ADMA belongs to the class of so-called uremic toxins, a group of compounds, which accumulate in the bloodstream during CKD and are associated with several adverse effects (Vanholder et al. 2003).

In contrast to ADMA, lower plasma concentrations of the structurally related and endogenously synthesized metabolite l-homoarginine are tightly correlated to a higher cardiovascular risk and an increase of all-cause mortality (März et al. 2010; Pilz et al. 2014b). l-Homoarginine is an endogenous, non-proteinogenic amino acid, which may increase nitric oxide availability and enhance endothelial function (Pilz et al. 2014a). The enzyme L-arginine:glycine amidinotransferase (AGAT), which is highly expressed in the kidney (Braissant et al. 2005), is one major enzyme of endogenous l-homoarginine synthesis (Choe et al. 2013). This was demonstrated by Genome Wide Association studies (GWAS), in which the presence of a function-modulating single-nucleotide polymorphism (SNP) in the gene encoding for the enzyme AGAT is associated with altered plasma concentrations of l-homoarginine (Choe et al. 2013). Interestingly, lower l-homoarginine plasma concentrations were found in patients with reduced kidney function (Tomaschitz et al. 2014). Because both enzymes (DDAH1 and AGXT2) degrading the uremic toxin ADMA as well as the enzyme AGAT which is of importance for l-homoarginine synthesis are located intracellularly, transport of both ADMA and l-homoarginine across the basolateral and luminal membrane of proximal tubule cells is crucial for the intracellular as well as plasma and urinary concentrations of these metabolites.

Transport proteins located in plasma membranes are generally important determinants of absorption, distribution and excretion of drugs and endogenous compounds (Shitara et al. 2005). Based on their transport direction, they are subdivided into two groups: uptake transporters mediate the uptake of substances from the extracellular space (e.g. blood) into cells, while export proteins mediate the transport of substances into the extracellular space. Most uptake transporters belong to the superfamily of SLC transporters (SLC = solute carriers) (Hediger et al. 2013), whereas export proteins are mostly members of the ABC transporter (ABC = ATP-binding cassette) superfamily. Unlike SLC transporters, ABC transporters depend on the hydrolysis of ATP as the main driving force to transport their substrates against a concentration gradient (König et al. 2013). In addition, some members of the SLC transporter superfamily are also able to mediate an export of compounds (Koepsell and Endou 2004; Masuda et al. 2006; Otsuka et al. 2005).

The process of urinary secretion is mediated by the combined action of a wide range of uptake transporters and export pumps that are expressed in the basolateral and luminal membrane of proximal tubule cells (Masereeuw et al. 2014). In human kidney, important uptake transporters located in the basolateral membrane of proximal tubule cells are the SLC21/SLCO family member OATP4C1 (gene symbol *SLCO4C1*) (Mikkaichi et al. 2004) and the SLC22 family members OCT2 (S*LC22A2*), OCT3 (*SLC22A3*), OAT1 (S*LC22A6*) and OAT2 (*SLC22A7*) (Koepsell 2013; Koepsell and Endou 2004). In addition, the SLC7 family member CAT1 (*SLC7A1*) is expressed in this membrane domain. ADMA has been characterized as low affinity substrate for CAT1 (Strobel et al. 2012) and OCT2 (Strobel et al. 2013). l-Homoarginine is also a substrate for CAT1 (Chafai et al. 2017).

In the luminal membrane, the ABC transporters P-glycoprotein (*ABCB1*) (del Moral et al. 1998), MRP2 (*ABCC2*) (Schaub et al. 1999) and MRP4 (*ABCC4*) (van Aubel et al. 2002) are expressed. Furthermore, the SLC47 family members MATE1 (*SLC47A1*) and MATE2-K (*SLC47A2*) are localized in this membrane domain (Masuda et al. 2006; Otsuka et al. 2005). ADMA and L-arginine were characterized as low affinity substrates of MATE1 (Strobel et al. 2013).

Human OATP4C1 is the only SLC21/SLCO family member expressed in human proximal tubule cells (Mikkaichi et al. 2004). Recently, we could demonstrate that both ADMA and l-homoarginine are substrates of OATP4C1. Furthermore, this transport protein is also capable of exporting ADMA and l-homoarginine out of cells (Taghikhani et al. 2019). Considering ADMA, these findings are in line with an animal study conducted by Toyohara et al., which investigated the effects of OATP4C1 overexpression on general cardiovascular outcome and uremic toxin plasma concentrations in the state of renal insufficiency (Toyohara et al. 2009). The authors found that after undergoing five-sixths nephrectomy (an established model to simulate renal failure), transgenic rats with overexpression of human OATP4C1 in the kidney showed reduced hypertension, cardiomegaly and inflammation in comparison to non-transgenic littermates. Interestingly, the plasma concentration of the uremic toxin ADMA was also reduced in OATP4C1 overexpressing rats. However, it is to date unknown, if an ABC transporter in the luminal membrane of proximal tubule cells is mediating the export of ADMA and l-homoarginine into urine and whether this acts differentially towards the two arginine derivatives.

To gain more insight into this topic, we established and characterized double-transfected MDCK cells expressing the uptake transporter OATP4C1 and the export pump P-glycoprotein (P-gp) simultaneously (MDCK-OATP4C1-P-gp). Using MDCK cell monolayers, we investigated the transcellular transport of ADMA and l-homoarginine in vectorial transport experiments, demonstrating that both substances are transported from the basolateral to the apical compartment of polarized grown MDCK-OATP4C1-P-gp cells to a considerably larger extent in comparison to the respective single-transfected cell lines (MDCK-OATP4C1, MDCK-P-gp) as well as the control cell line (MDCK-VC).

## Methods

### Chemicals

[^3^H]labeled imatinib-mesylate (53,9 mCi/mmol) and unlabeled imatinib-mesylate were purchased from Novartis (Basel, Switzerland).[^3^H]labeled digoxin (20 Ci/mmol) was from American Radiolabeled Chemicals (St.Louis, USA).[^3^H]labeled ADMA (25 Ci/mmol) was purchased from Biotrend (Cologne, Germany), [^3^H]labeled l-homoarginine (6 Ci/mmol) was from ViTrax (St. Louis, USA) and [^3^H]inulin was obtained from PerkinElmer Life Science GmbH (Rodgau-Jügesheim, Germany). Unlabeled ADMA was obtained from Enzo Life Sciences (Lörrach, Germany) and unlabeled l-homoarginine was from Arcos Organics (New Jersey, USA). Dulbecco’s phosphate buffered saline, minimum essential medium (MEM), fetale bovine serum (FBS) and 0.05%-trypsin–EDTA were obtained from Life Technologies (Paisley, UK). Geneticin disulfate (G418), hygromycin B and penicillin–streptomycin were purchased from Invitrogen GmbH (Paisley, UK). Preparation of cells for immunofluorescence analysis was conducted with Immu-Mount purchased from Thermo Fisher Scientific (Rockford, USA). Sodium butyrate was from Merck KGaA (Darmstadt, Germany). BCA Pierce Protein Assay Kit was purchased from Life Technologies GmbH. Transwell membrane inserts (14 mm diameter, 0.4 µm pore size) and 12-well culture plates were from Greiner Bio-One (Frickenhausen, Germany). Unless stated otherwise, all other chemicals and reagents were obtained from Carl Roth (Karlsruhe, Germany) and Sigma-Aldrich (Munich, Germany) in the highest available purity.

### Cell culture

Stably transfected MDCK-VC, MDCK-OATP4C1 and MDCK-P-gp cells as well as the new established double-transfected MDCK-OATP4C1-P-gp cells were cultivated under conditions as previously described (Misaka 2016). Cultivation was conducted using MEM supplemented with 10% FBS, 100 U/ml penicillin and 100 µg/ml streptomycin at 37 °C and 5% CO_2_. For selective cell cultivation, either 800 µg/ml G418 or 250 µg/ml hygromycin B were added to the medium, depending on the restriction gene of the respective MDCK cell line. For cultivation of MDCK-OATP4C1-P-gp double-transfectants, both G418 and hygromycin B were applied to the medium. All cells were routinely subcultured twice a week using trypsin (0.05%)-EDTA (0.02%) solution.

### Generation of stably transfected MDCK cells

Cloning of the human *SLCO4C1* cDNA encoding human OATP4C1 and establishment of the expression vector pOATP4C1.31 has been described recently (Taghikhani et al. 2019). Generation of MDCK-VC and MDCK-OATP4C1 cells was conducted according to previously published protocols (Misaka et al. 2016; Taghikhani et al. 2017). MDCK-P-gp cells were from the University of Greifswald (Dr. M. Keiser, Center of Drug Absorption and Transport). To generate a double-transfected MDCK-OATP4C1-P-gp cell line, MDCK-P-gp cells were transfected with the pOATP4C1.31 plasmid using the Effectene™ Transfection Reagent Kit (QIAGEN GmbH, Hilden, Germany) according to the manufacturer’s instructions. For selection of successfully double-transfected cells, cultivation was conducted in medium supplemented with 800 µg/ml G418 [to select for OATP4C1] and 250 µg/ml hygromycin B [to select for P-gp] for 4 weeks. Cells were then screened for mRNA expression of *SLCO4C1* and *ABCB1* cDNA using a LightCycler-based qRT-PCR approach as described (Taghikhani et al. 2019). The clone with the highest *SLCO4C1* mRNA expression was further characterized for protein expression of OATP4C1 and P-gp by immunoblot and immunofluorescence analysis.

### Immunoblot analysis

Isolation of total protein from MDCK-VC, MDCK-OATP4C1, MDCK-P-gp and MDCK-OATP4C1-P-gp cells as well as subsequent detection of target proteins by immunoblot analysis were conducted as described previously (Taghikhani 2019). For detection of P-gp and OATP4C1, 30 µg of protein isolate were diluted with Laemmli buffer and incubated for 30 min at 37 °C. Protein separation was conducted using 7.5% SDS–polyacrylamide gels. After separation, proteins were transferred to nitrocellulose membranes. To detect OATP4C1, the membrane was incubated with a 1:1 000-dilution (in 0.1% PBS Tween 20 containing 5% skim milk) of the polyclonal rabbit anti-human OATP4C1 AVV antiserum (Taghikhani et al. 2019). A 1:10 000 diluted goat anti-rabbit IgG conjugated with horseradish peroxidase (GE Healthcare Life Sciences, Buckinghamshire, UK) was used as secondary antibody. To detect P-gp, the monoclonal mouse anti-human P-gp antibody MDR-1 (1:4 000; Sigma Aldrich GmbH) was applied to the membrane, followed by an incubation with peroxidase-labeled goat anti-mouse IgG (1:2 000, Dianova GmbH, Hamburg, Germany). Protein signals were detected using Clarity™ ECL Western Blotting Substrate (Bio-Rad Laboratories Inc., Hercules, USA). For control purposes, membranes were stripped and reincubated with a monoclonal mouse anti-human β-actin primary antibody (Sigma-Aldrich, St. Louis, USA, 1:10 000 dilution) and a goat anti-mouse IgG antibody.

### Confocal laser scanning immunofluorescence microscopy

The localization of the recombinantly overexpressed proteins in the stably transfected MDCK cell lines was studied by immunofluorescence microscopy. An initial amount of 5 × 10^5^ cells/well was cultivated in Transwell membrane inserts (14 mm diameter, 0.4 µm pore size; Greiner Bio-One, Frickenhausen, Germany) for 48 h. Afterwards, cells were induced by aspirating the medium on top of the cells and replacing it with fresh medium supplemented with 10 mM sodium butyrate (Cui et al. 1999). After 24 h of further cultivation, cells were treated with ice-cold methanol solution (70% v/v) and permeabilized by applying a TBS-solution containing 0.4% triton for 10 min at RT. Then, cells were blocked with 2% BSA solution and incubated with either polyclonal rabbit anti-human OATP4C1 AVV antiserum (1:500 diluted in 2% BSA solution) or monoclonal anti-human P-gp antibody MDR-1 (1:2 000) over night at 4 °C. As secondary antibody for OATP4C1, a goat anti-rabbit IgG conjugated with Alexa Fluor 568 (Invitrogen GmbH, Karlsruhe) was used. For detection of P-gp Cy2-conjugated goat, anti-mouse IgG (Dianova GmbH, Hamburg) was applied. Cells were incubated with the respective secondary antibody for 30 min at RT. For simultaneous localization of OATP4C1 and P-gp in MDCK-OATP4C1-P-gp double-transfectants, both primary antibodies as well as the respective secondary antibodies were incubated with the cells. After application of antibodies, the membranes with the cells were cut out and mounted on microscope slides using Immu-Mount medium. Immunofluorescence analysis was conducted using an Axiovert 100 M confocal laser scanning microscope (Carl Zeiss Micro Imaging GmbH, Jena, Germany) and version 4.2.0.121 of the Zeiss LMS Image Browser.

### Transcellular transport studies with stably transfected MDCK cells

Transport activity of P-gp in MDCK-P-gp cells was verified by analyzing the time-dependent transcellular transport of the P-gp substrate imatinib (5 µM). Transcellular transport in MDCK-OATP4C1-P-gp double-transfectants and respective MDCK-OATP4C1 and MDCK-P-gp single-transfectants as well as in the MDCK-VC control cell line was analyzed as previously described (Taghikhani et al. 2017). Cells were cultured in Transwell membrane inserts (14 mm diameter, 0.4 µm pore size; Greiner Bio-One, Frickenhausen, Germany) in an initial density of 5 × 10^5^ cells/well. Cells were cultivated at 37 °C for 48 h before inducing them by replacing the medium with fresh medium containing 10 mM of sodium butyrate. After 24 h of further incubation, cells were used for transcellular transport studies.

To study the transcellular transport, cells were washed with pre-warmed (37 °C) uptake buffer applied to both the basolateral and apical compartment. The buffer was aspirated and another 800 µl of fresh, warm uptake puffer were applied to the apical compartment. The respective radiolabeled substrate in the desired concentration was applied to the basolateral compartment of the MDCK cell monolayer (“donor solution”). After incubating the cells for stated time points, the amount of radioactivity on the apical compartment was measured by liquid scintillation counting.

After establishment of MDCK-OATP4C1-P-gp double-transfectants, the combined activity of OATP4C1 and P-gp was verified by analyzing time-dependent transcellular transport of digoxin (5 µM), a described substrate of both OATP4C1 and P-gp (Fromm et al. 1999; Mikkaichi et al. 2004) in comparison to MDCK-VC, MDCK-OATP4C1 and MDCK-P-gp cells. To study time-dependent transcellular transport, 80 µl of uptake buffer were removed from the apical compartment (supernatant) of the cells during incubation at designated time points (1, 2, 3 and 4 h) and prepared for liquid scintillation counting by adding 4 ml of Ultima Gold XR (PerkinElmer LAS GmbH, Rodgau-Jügesheim). Every time the removed volume was immediately replaced with 80 µl of pre-warmed fresh uptake buffer. After incubation time has elapsed, membranes were cut out and the cells on top of the membranes were lysed using 600 µl of a 0.2% SDS solution. The protein concentration of the lysate was measured by BCA Assay according to the manufacturer`s instructions.

Concentration dependency of transcellular transport of ADMA and l-homoarginine was studied by applying the respective compound in concentrations of 1, 20, 50 and 100 µM on the basolateral compartment of MDCK transfectants. Cells were incubated with the compounds for 1 h at 37 °C. Subsequently, 400 µl of uptake buffer were taken from the apical side (supernatant) and prepared for scintillation counting. Cells were lysed and the cellular protein content was quantified as described. 400 µl (or 80 µl for time-dependency analysis) of the applied donor solutions were also analyzed by scintillation counting as reference. To investigate the transcellular leakage, cells were routinely treated likewise with 50 µM [^3^H]inulin.

### Data analysis and statistical analysis

The data depicted in the figures are expressed as means ± S.E.M. Unless stated otherwise, statistical analysis was conducted using one-way ANOVA Bonferroni multiple comparison test. Statistical significance was attributed at a *p* value of < 0.05 (*p). The amount of radioactivity measured in the apical compartment was normalized to the amount of radioactivity in the respective donor solution and the protein content of the cells in the Transwell membrane insert. Unless stated otherwise, transcellular transport was studied in two separate experiments each performed in triplicates (*n* = 2 × 3 i.e. *n* = 6). Data were analyzed using GraphPad Prism (Version 5.01, 2007, GraphPad Software, San Diego, CA, USA).

## Results

### Characterization of the double-transfected MDCK-OATP4C1-P-gp cell line

**Fig. 1:**
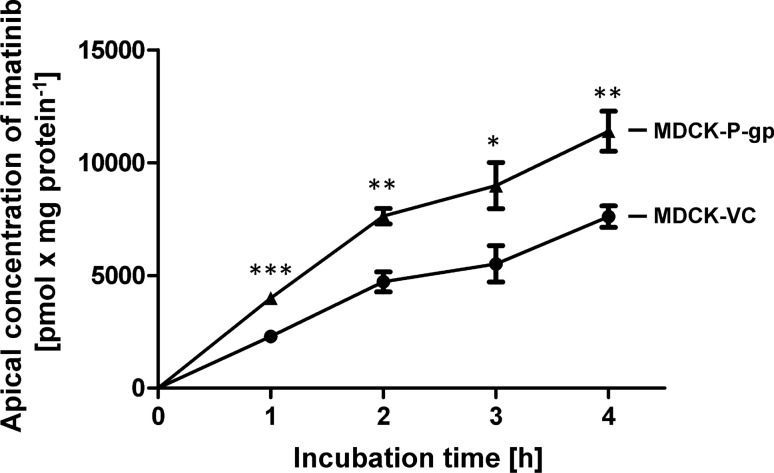
Time-dependent transcellular, basal to apical (b-a) transport of imatinib across MDCK cell monolayers. Imatinib (5 µM) was applied to the basolateral compartment of MDCK-VC cells (which served as control cell line) and MDCK-P-gp cells with stable overexpression of human P-gp. Transcellular transport was quantified by measuring the amount of imatinib appearing in the apical compartment after 1, 2, 3 and 4 h of incubation. Data are expressed as means ± S.E.M. (*n* = 2 × 2 i.e. *n* = 4). *p < 0.05, **p < 0.01, ***p < 0.001 vs. MDCK-VC; unpaired, two-tailed t-test

**Fig. 2:**
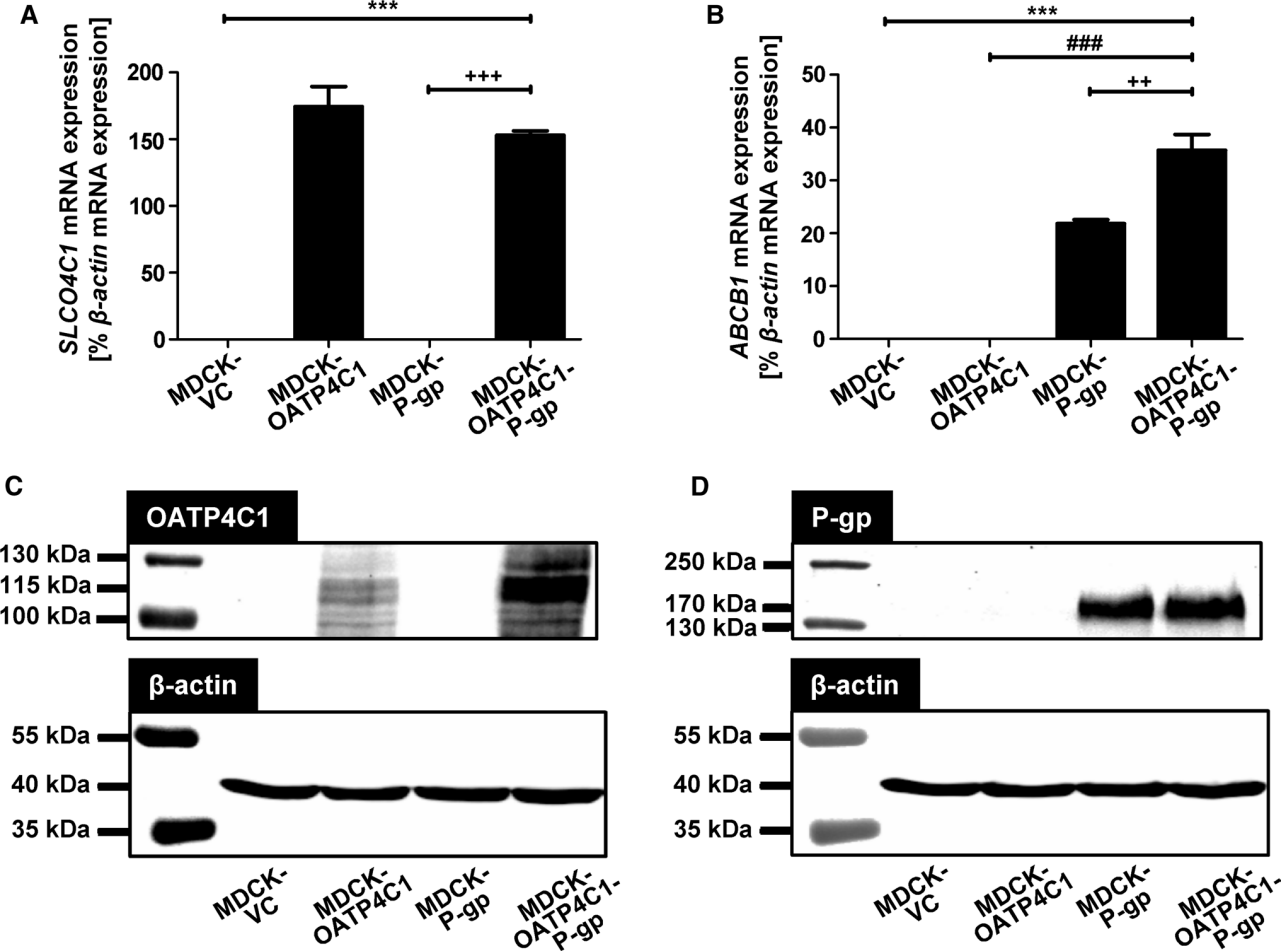
Characterization of single-transfected MDCK-OATP4C1 and MDCK-P-gp cells, double-transfected MDCK-OATP4C1-P-gp cells and the control cell line MDCK-VC. qRT-PCR analysis of *SLCO4C1* (encoding human OATP4C1) mRNA expression (**a**) and *ABCB1* (encoding human P-gp) mRNA expression (**b**). The mRNA expression is reported relative to the expression of the housekeeping gene β-actin. Immunoblot analysis of OATP4C1 expression (**c**) and P-gp expression (**d**) in the stably-transfected MDCK cell lines. MDCK-VC = control cell line, MDCK-OATP4C1 = MDCK cell line recombinantly overexpressing human OATP4C1, MDCK-P-gp = MDCK cell line recombinantly overexpressing human P-gp, MDCK-OATP4C1-P-gp = double-transfected MDCK cell line simultaneously overexpressing human OATP4C1 and human P-gp. Staining of β-actin served as loading control. ***p < 0.001 vs. MDCK-VC; ^###^*p* < 0.001 vs. MDCK-OATP4C1; ^++^*p* < 0.01, ^+++^*p* < 0.001 vs. MDCK-P-gp; one-way ANOVA Bonferroni multiple comparison test

To check for proper P-gp transport activity in the MDCK-P-gp single-transfected cells used for generation of the double-transfected MDCK-OATP4C1-P-gp cells, transcellular transport of the P-gp substrate imatinib was analyzed (Fig. 1). At all studied time points (1, 2, 3 and 4 h), the amount of imatinib in the apical compartment of MDCK-P-gp cells significantly exceeded the amount measured in MDCK-VC control cells. This confirms proper P-gp transport activity in MDCK-P-gp single-transfected cells. Subsequently, these cells were stably transfected with the plasmid pOATP4C1.31 resulting in a double-transfected MDCK cell line with recombinant overexpression of OATP4C1 and P-gp. Analysis of mRNA expression by qRT-PCR, quantified related to the expression of the housekeeping gene β-actin (100%) showed that *SLCO4C1* mRNA expression was 174.4 ± 15.1% in MDCK-OATP4C1 single-transfectants and 152.9 ± 3.4% in MDCK-OATP4C1-P-gp double-transfectants (Fig. 2a). The respective mRNA expression of *ABCB1* was 21.8 ± 0.7% in MDCK-P-gp single-transfected cells and 35.7 ± 3.0% in MDCK-OATP4C1-P-gp double-transfectants (Fig. 2b). OATP4C1 and P-gp protein expression was successfully verified by immunoblot analysis (Fig. 2c, d). In line with published data for OATP4C1 (Taghikhani 2019; Mikkaichi et al. 2004; Shirasaka 2009), a band with a molecular weight of 115 kDa was detected in the MDCK-OATP4C1 single-transfected as well as in the MDCK-OATP4C1-P-gp double-transfected cell line (Fig. 2c). For P-gp, a band with a molecular mass of 170 kDa was detected in MDCK-P-gp and MDCK-OATP4C1-P-gp cells (Fig. 2d) (Shirasaka 2009).

**Fig. 3:**
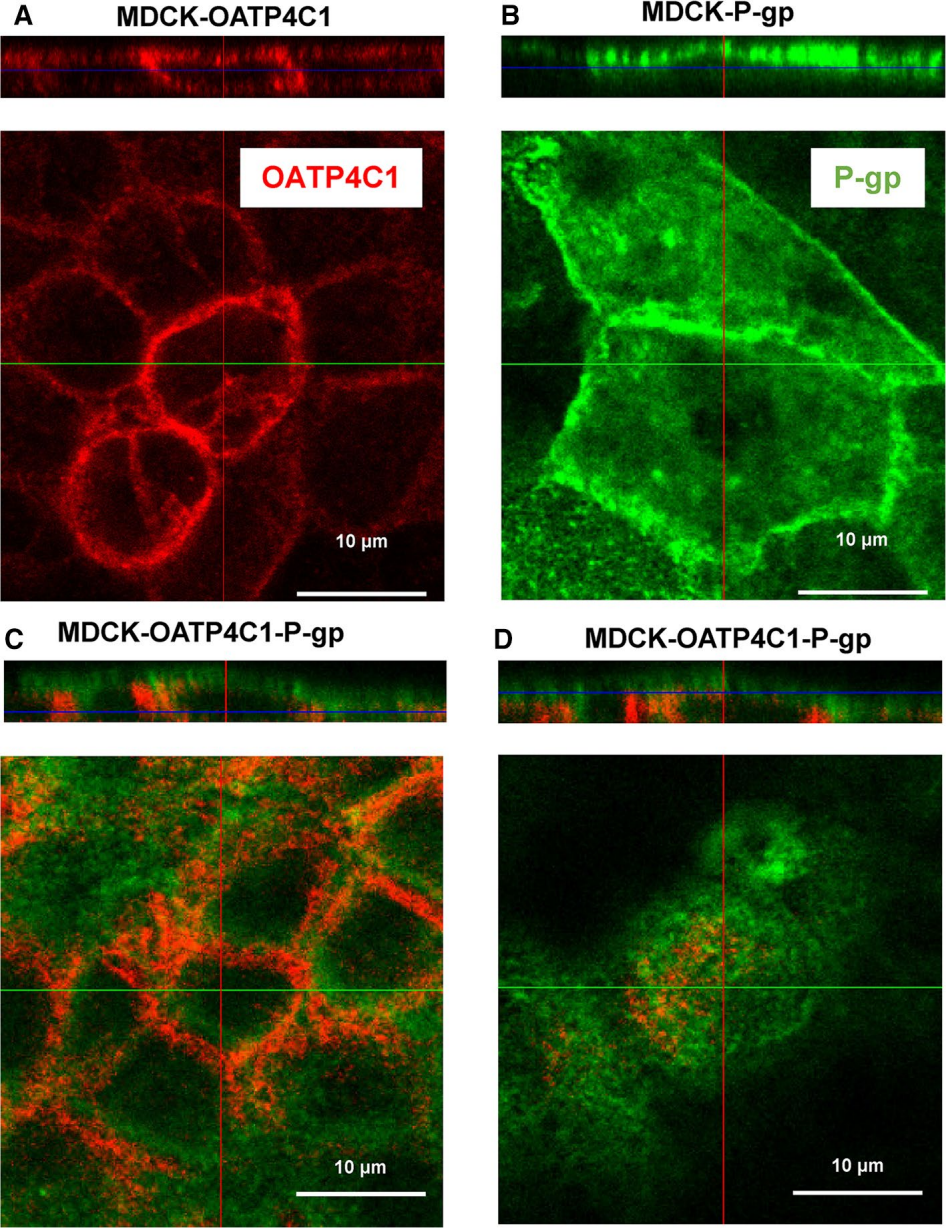
Localization of OATP4C1 and P-gp in MDCK cells. OATP4C1 was localized using a Alexa Fluor 568-conjugated secondary antibody (red color). For P-gp, a Cy2-conjugated antibody was used (green). **a**, **c** The localization of OATP4C1 in single-transfected MDCK-OATP4C1 cells (**a**) and double-transfected MDCK-OATP4C1-P-gp cells, **b**, **d** the localization of P-glycoprotein in single-transfected MDCK-P-gp cells (**b**) and double-transfected MDCK-OATP4C1-P-gp cells

Localization of the recombinantly expressed proteins was studied by immunofluorescence analysis. The results demonstrate the basolateral localization of OATP4C1 in MDCK-OATP4C1 and MDCK-OATP4C1-P-gp cells (red fluorescence in Fig. 3a,c) and the apical localization of P-gp in MDCK-P-gp and MDCK-OATP4C1-P-gp cells (green fluorescence in Fig. 3b, d).

**Fig. 4:**
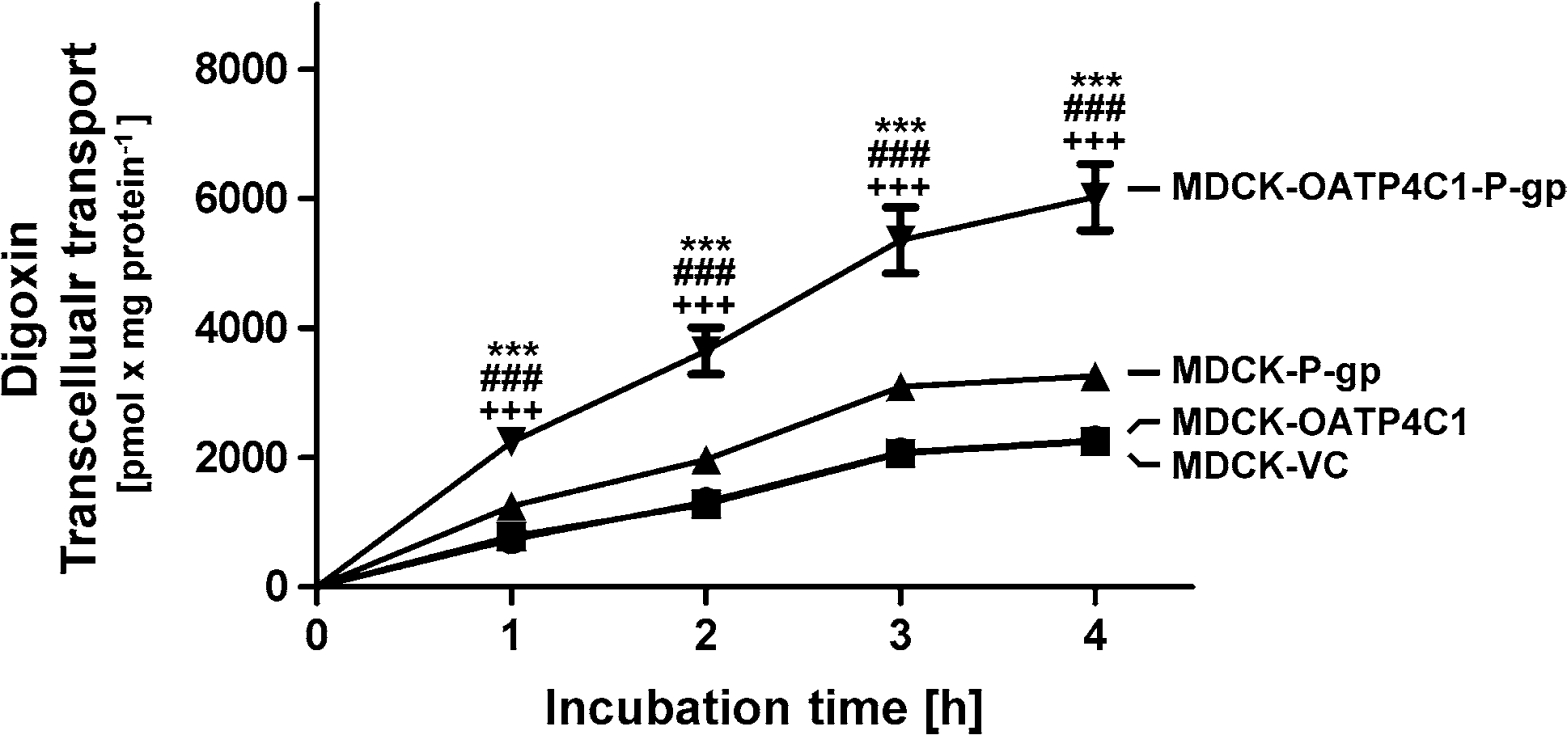
Time-dependent transcellular, basal to apical (b-a) transport of digoxin (5 µM) across MDCK cell monolayers. Digoxin was applied on the basolateral side of MDCK cells. Transcellular transport was quantified by measuring the amount of digoxin in the apical compartment after 1, 2, 3 and 4 h of incubation. Data are expressed as means ± S.E.M. (*n* = 2 × 3 i.e. *n* = 6) **p < 0.01, ***p < 0.001 vs. MDCK-VC; ^##^*p* < 0.01, ^###^*p* < 0.001 vs. MDCK-OATP4C1; ^++^*p* < 0.01, ^+++^*p* < 0.001 vs. MDCK-P-gp; one-way ANOVA Bonferroni multiple comparison test

Finally, transport function of OATP4C1 and P-gp was explored using the common OATP4C1 and P-gp substrate digoxin. Using 5 µM digoxin in transcellular transport experiments (Fig. 4) significantly more digoxin was transported from the basolateral to the apical compartment in MDCK-OATP4C1-P-gp double-transfected cells compared to single-transfectants and the respective MDCK-VC cells. Transport ratios of 5 µM digoxin are summarized in Table 1. In comparison to the MDCK-VC control cell line a 1.7-fold higher transcellular digoxin transport was observed in MDCK-P-gp single-transfectants after an incubation time of 1 h. This transport ratio is in line with data reported in previous studies (Taipalensuu et al. 2004; Tang et al. 2002), which examined the transport of digoxin in P-gp overexpressing MDCK cells.

**Table 1:** Vectorial transport ratios for digoxin based on time-dependency experiments (Fig. 4)

	Average ratios of vectorial digoxin transport (relative to MDCK-VC cells)			
	1 h	2 h	3 h	4 h
MDCK-OATP4C1	1.08	0.97	0.99	0.99
MDCK-P-gp	1.72	1.50	1.49	1.44
MDCK-OATP4C1-P-gp	3.07	2.77	2.57	2.66

Digoxin was added to the basal compartment of the respective MDCK cell line in a concentration of 5 µM. The amount of digoxin in the apical compartment was calculated after 1, 2, 3 and 4 h of incubation

### Concentration and time dependency of transcellular transport of ADMA and l-homoarginine in MDCK-OATP4C1-P-gp double-transfectants

**Fig. 5:**
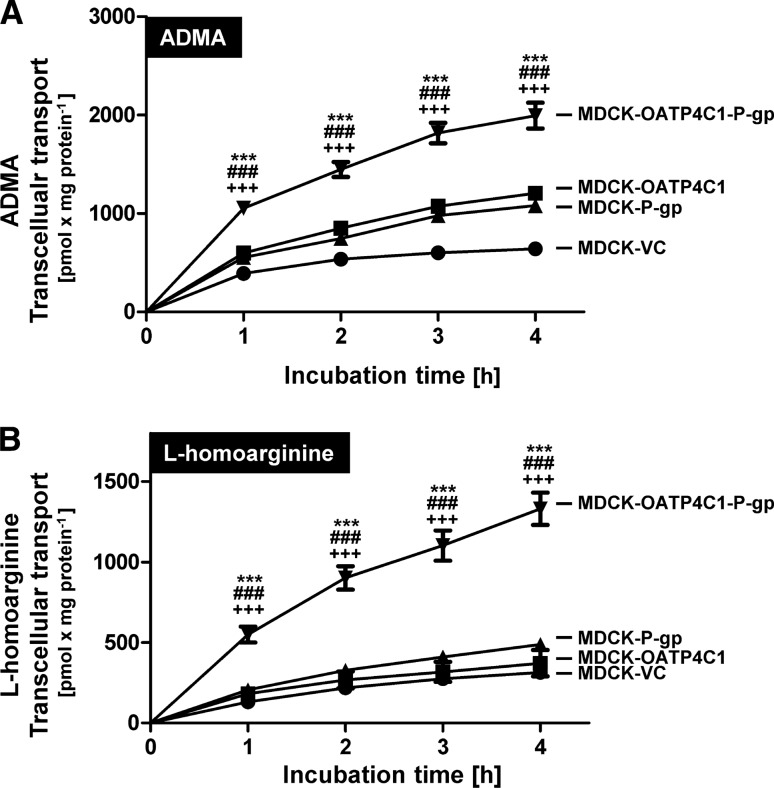
Time-dependent transcellular, basal to apical (b-a) transport of ADMA (**a**) and l-homoarginine (**b**) across MDCK cell monolayers. One µM of compound was applied to the basolateral compartment of polarized grown MDCK cells. Transcellular transport was quantified by measuring the amount of ADMA or l-homoarginine in the apical compartment after 1, 2, 3 and 4 h. Data are expressed as means ± S.E.M. (*n* = 2 × 3 or *n* = 6). ***p < 0.001 vs. MDCK-VC;  ^###^*p* < 0.001 vs. MDCK-OATP4C1, ^+++^*p*<0.001 vs MDCK-P-gp; one-way ANOVA Bonferroni multiple comparison test

Time dependency experiments demonstrated that at all examined time points (1, 2, 3 and 4 h) transcellular transport of 1 µM ADMA (Fig. 5a) and of 1 µM l-homoarginine (Fig. 5b) in MDCK-OATP4C1-P-gp cells was significantly higher compared to the transport rates in the other tested cell lines.

**Fig. 6:**
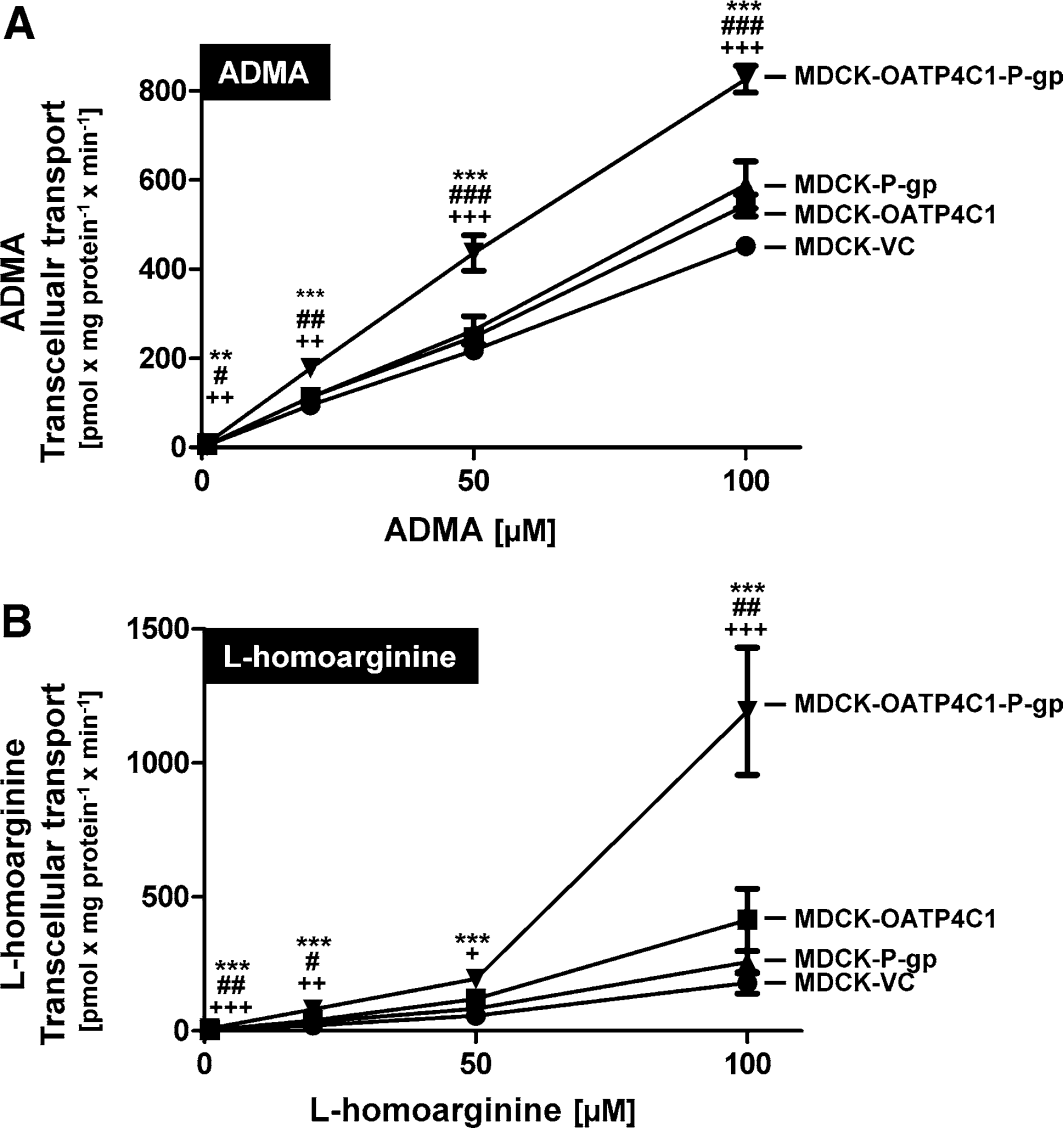
Concentration-dependent transcellular, basal to apical (b-a) transport of ADMA (**a**) and l-homoarginine (**b**) across MDCK cell monolayers. ADMA and l-homoarginine were added in concentrations of 1, 20, 50 and 100 µM to the basolateral compartment of polarized grown MDCK cells. Transcellular transport was quantified by measuring the amount of ADMA or l-homoarginine in the apical compartment after 1 h. Data are expressed as means ± S.E.M. (*n* = 2 × 3 i.e. *n* = 6). **p < 0.01, ***p < 0.001 vs. MDCK-VC; ^#^*p* < 0.05, ^##^*p* < 0.01, ^###^*p* < 0.001 vs. MDCK-OATP4C1; ^+^*p* < 0.05, ^++^*p* < 0.01, ^+++^*p* < 0.001 vs. MDCK-P-gp; one-way ANOVA Bonferroni multiple comparison test

In concentration-dependency experiments, transcellular transport of ADMA (Fig. 6a) and l-homoarginine (Fig. 6b) was significantly higher in MDCK-OATP4C1-P-gp double-transfected cells compared to single-transfected and control cells over the entire concentration range. At the lowest tested ADMA concentration of 1 µM, a transport rate of 9.2 ± 1.2 pmol × mg protein^−1^ × min^−1^ was detected in MDCK-OATP4C1-P-gp cells compared to a transport rate of 4.8 ± 0.4 pmol × mg protein^−1^ × min^−1^ in MDCK-VC cells (transport ratio: 1.92-fold). For 20 µM, 50 µM and 100 µM ADMA, transport ratios of 1.87, 2.00 and 1.83 were calculated, respectively.

Using the same substrate concentrations for l-homoarginine, transport ratios of 3.44 (for 1 µM), 3.94 (for 20 µM), 3.41 (for 50 µM) and 6.65 (for 100 µM) were determined. A detailed summary of the transport ratios of all cell lines relative to the vectorial transport in MDCK-VC cells is presented in Table 2 (ADMA) and Table 3 (l-homoarginine).

**Table 2:** Vectorial transport ratios for ADMA based on concentration-dependency experiments (Fig. 6)

	Average ratios of vectorial ADMA transport (relative to MDCK-VC cells)			
	1 µM	20 µM	50 µM	100 µM
MDCK-OATP4C1	1.17	1.19	1.14	1.20
MDCK-P-gp	1.10	1.19	1.20	1.30
MDCK-OATP4C1-P-gp	1.92	1.87	2.00	1.83

ADMA was added to the basal compartment of the respective MDCK cell line and the amount of ADMA in the apical compartment was calculated after 1 h of incubation

**Table 3:** Vectorial transport ratios for l-homoarginine based on concentration-dependency experiments (Fig. 6)

	Average ratios of vectorial l-homoarginine transport (relative to MDCK-VC cells)			
	1 µM	20 µM	50 µM	100 µM
MDCK-OATP4C1	1.33	1.92	2.09	2.31
MDCK-P-gp	1.11	1.61	1.45	1.43
MDCK-OATP4C1-P-gp	3.44	3.94	3.41	6.65

l-Homoarginine was added to the basal compartment of the respective MDCK cell line and the amount of l-homoarginine in the apical compartment was calculated after 1 h of incubation

## Discussion

The aim of this study was to gain more insights into the role of transport proteins involved in the homeostasis and renal elimination of arginine derivatives. Therefore, we established a double-transfected MDCK cell line with simultaneous expression of the basolaterally localized transport protein OATP4C1 and the apically localized export pump P-gp (MDCK-OATP4C1-P-gp). After successfully establishing and characterizing this new cell line, vectorial transport of the arginine derivatives ADMA and l-homoarginine was studied in MDCK cell monolayers. The transcellular transport experiments demonstrated that MDCK-OATP4C1-P-gp cells show a considerably higher transcellular transport of both ADMA and l-homoarginine in comparison to the respective single-transfected cell lines (MDCK-OATP4C1, MDCK-P-gp) and the control cell line (MDCK-VC).

For ADMA, it is known that a combination of renal filtration, tubular secretion, tubular reabsorption and degradation by the enzyme DDAH1 in proximal tubule cells result in the net renal excretion of ADMA (Nijveldt et al. 2002). For ADMA secretion, CAT1-, OCT2- (Strobel et al. 2013) or OATP4C1-mediated (Taghikhani et al. 2019) uptake from blood into proximal tubule cells is required. Approximately, 80% of ADMA taken up into proximal tubule cells is metabolized e.g. by the enzyme DDAH1 with the remaining 20% being excreted unchanged into urine (Kielstein et al. 2009). These results suggested that an export pump or an export protein located in the luminal membrane of proximal tubule cells might be involved in this export. ADMA has been characterized as a possible substrate for the SLC47 family member MATE1 located in this membrane domain, but tested so far only in uptake experiments using HEK293 cells recombinantly overexpressing MATE1 (Strobel et al. 2013). However, MATE1 normally acts as an export protein and it has been demonstrated with single-transfected HEK-MATE1 cells and double-transfected MDCK-OCT2-MATE1 cells that some substrates could be exported by MATE1 without being taken up by the same transport protein (Müller et al. 2017). This suggests different transport characteristics for MATE-mediated uptake or export and MATE1-mediated export of ADMA has not been demonstrated so far. In addition to MATE1, P-gp has been localized to the luminal membrane of proximal tubule cells (del Moral et al. 1998) and our results with the double-transfected MDCK-OATP4C1-P-gp cells indicated that P-gp is involved in the renal secretion of the uremic toxin ADMA.

In contrast to ADMA, urinary concentrations of l-homoarginine in humans are low and almost no renal elimination has been measured (Frenay et al. 2015) possibly due to efficient reabsorption. This suggests that several transport proteins in the luminal and basolateral membrane domain of proximal tubule cells are necessary for the renal handling of this arginine derivative. So far, l-homoarginine has been characterized as substrate for the SLC7 family members CAT1, CAT2A and CAT2B with CAT1 being expressed in proximal tubule cells. For l-homoarginine, a *K*_m_ value of 175 µM has been calculated for CAT1-mediated uptake (Chafai et al. 2017). In addition, l-homoarginine is also a substrate of the SLC21/SLCO family member OATP4C1 located in the same membrane domain with a K_m_ value of 49.9 µM (Taghikhani et al. 2019). A transport protein located in the luminal membrane of proximal tubule cells mediating the reabsorption of filtrated l-homoarginine (or of its precursor L-arginine) has not been characterized so far. Due to the fact, that l-homoarginine is synthesized in proximal tubule cells by the enzyme AGAT (Braissant et al. 2005), a transport protein in the basolateral membrane is necessary for the export of l-homoarginine into the systemic circulation. We have recently provided evidence that OATP4C1 is also able to mediate the export of l-homoarginine (Taghikhani et al. 2019). Data presented in this study indicate that l-homoarginine is also a substrate of the export pump P-gp located in the luminal membrane. The potential importance of P-gp-mediated l-homoarginine export could be in the maintenance of intracellular l-homoarginine homoeostasis in proximal tubule cells.

Taken together, the results presented in this manuscript demonstrated for the first time, that the arginine derivatives ADMA and l-homoarginine may be substrates of the export pump P-gp, which is localized in the luminal membrane of proximal tubule cells. Because both enzymatic degradation of ADMA and enzymatic synthesis of l-homoarginine take place in proximal tubule cells, P-gp together with the basolaterally localized transport protein OATP4C1 may be important for the renal transport and the intracellular homeostasis of these substances. Under normal physiological conditions, l-homoarginine is synthesized in proximal tubule cells and subsequently exported by OATP4C1 into the systemic circulation, whereas ADMA is transported by OATP4C1 (and CAT1) from the bloodstream into the proximal tubule cells, where it is being degraded by e.g. DDAH1 or directly transported by P-gp or MATE1 into urine. The precise role and relative contribution of P-gp to the urinary secretion of l-homoarginine in humans (e.g. under conditions of high food intake) has to be elucidated in the future.
